# Long‐term results of brentuximab vedotin and bendamustine for first‐line treatment of Hodgkin lymphoma in the elderly (HALO trial)

**DOI:** 10.1111/bjh.70763

**Published:** 2026-08-26

**Authors:** Andrea Gallamini, Simonetta Viviani, Davide Rapezzi, Caterina Patti, Pierre Feugier, Alessandro Rambaldi, Fontanet Bijou, Anna Borra, Cécile Borel, Tanguy Pace‐Loscos, Renaud Schiappa, Stephane Chauvie, Colin Debaigt, Anna Guidetti, Olivier Humbert, Jean‐Marc Schiano De Colella

**Affiliations:** ^1^ Onco‐Hematology, Centre Antoine‐Lacassagne Nice France; ^2^ Division of Onco‐Hematology IEO, European Institute of Oncology, IRCCS Milano Italy; ^3^ Hematology Santa Croce Hospital Cuneo Italy; ^4^ Hematology and BMT Unit Ospedali Riuniti Villa Sofia e Cervello Palermo Italy; ^5^ Hematology CHU Brabois Nancy France; ^6^ Medical Oncology Research Foundation Bergamo Italy; ^7^ Hematology Bergonié Institute Bordeaux France; ^8^ Medical Oncology University Institute of Oncology Oncôpole Toulouse France; ^9^ Department of Epidemiology, Biostatistics and Health Data, Centre Antoine Lacassagne University of Côte d'Azur Nice France; ^10^ Medical Physics Department Santa Croce Hospital Cuneo Italy; ^11^ Department of Clinical Research and Innovation, Centre Antoine Lacassagne University of Côte d'Azur Nice France; ^12^ Department of Hematology and Stem Cell Transplantation Fondazione IRCCS Istituto Nazionale dei Tumori Milano Italy; ^13^ Nuclear Medicine Department Centre Antoine Lacassagne University of Côte d'Azur Nice France; ^14^ BMT Unit, Hematology Paoli Calmette Institute Marseille France

**Keywords:** bendamustine, brentuximab vedotin, elderly, Hodgkin lymphoma

## Abstract

The Hodgkin lymphoma treatment in the elderly (eHL) is a challenge due to a narrow therapeutic window between therapy effectiveness and toxicity. Both bendamustine (Be) and brentuximab vedotin (BV) are well‐tolerated, effective drugs in relapsing HL, but no data exist on Be‐BV frontline treatment in eHL. The prospective, open‐label, phase I/II Hodgkin lymphoma combining Adcetris® and Levact® in Old patients (HALO) study was launched (Clinical Trial.Gov Id. 02467946) to test safety and efficacy of Be‐BV in untreated eHL. The mean age was 70.75 (62–79), stage IIB in 12, III in 14 and IV in 31 patients. Most had B‐symptoms and an International Prognostic Score (IPS) ≥3 (39). Despite a good performance status and both a high activity of daily living (ADL) (≥6 in 85%) and instrumental activities of daily living (IADL) scores (≥8 in 77%), 68% of patients had ≥5 comorbidities, with a Cumulative Illness Rating Scale‐Geriatric (CIRS)‐G score >3 in 75%. A complete metabolic response (CMR) was achieved in 44/57 of patients. The 6‐year overall survival (OS) and progression‐free survival (PFS) in per‐protocol (PP) analysis were 67% (95% confidence intertvals [CI] 53–84) and 45% (95% CI 32–65) respectively. A PFS event was observed in 35: 13 progressions (10 deaths), 13 relapses (6 deaths) and 9 deaths. Be‐BV, when given in a full dose schedule, is an effective regimen for unselected, poor‐risk elderly Hodgkin lymphoma (HL) patients.

## INTRODUCTION

Classical Hodgkin lymphoma (cHL) is considered a curable cancer, with an overall survival and disease‐free survival rate of 90% and 80% respectively.^1^

Nonetheless, the 5‐ and 10‐year survival rate of patients aged more than 60 does not exceed 60% and 40%, respectively.^2^, ^3^ There is indeed evidence that cHL in the elderly is a different disease compared to adults,^4^ thus hampering a direct comparison between both age subsets. The International Prognostic Score (IPS) is not helpful in the aged, as it was originally designed for patients aged up to 65 years.^5^ The differences in pathogenetic mechanisms,^6^ histopathology^7^, ^8^ and clinical presentation^9^ underpin the different outcomes in both age brackets. Other important factors with a negative impact on treatment outcome are comorbidities,^10^ frailty^11^, ^12^ and anthracycline‐free chemotherapy.^1^

Brentuximab vedotin (BV) in combination with bendamustine (Be), followed by autologous haematopoietic stem cell transplantation, proved very effective in achieving long‐term disease control in relapsed/refractory cHL in adults.^13^

In the international, multicentre, randomized phase 3 Echelon‐1 clinical trial in advanced‐stage (III‐IV) cHL, the A‐Doxorubicine‐Vinblastine‐Dacarbazine chemotherapy schedule (AVD) regimen with BV at the dose of 1.8 mg/m^2^ on days 1 and 8, replacing bleomycin in the classical Doxorubicin, Bleomycin, Vinblastine and Dacarbazine (ABVD) backbone, proved superior to standard ABVD in long‐term disease control, both in terms of 6‐year progression‐free survival (PFS) and overall survival (OS).^14^, ^15^ However, compared to adult, this advantage did not persist overtime in the elderly (>60 years) patients: the modified progression‐free survival (mPFS) after 2 and 5 years was roughly similar in both arms (A + AVD: 70.3%–67.1% vs. ABVD: 71.4%–61.6%).^14^, ^15^ Later, Evens reported the results of a phase 2 study in untreated cHL elderly patients (60–88 years.) including sequential administration of BV (1.8 mg/kg. two doses) followed by four AVD cycles, and four more doses of BV. This new regimen proved very effective, achieving up to 90% of complete remissions in a cohort of 28/48 (58%) patients who were able to conclude the entire regimen, while in 20 patients (42%) BV was discontinued for neurotoxicity.^16^

Based on these preliminary reports, in 2015 we launched a prospective multicentre single‐arm phase 1/2 clinical trial aimed at assessing the feasibility and the efficacy of the combined administration of Be‐BV in untreated advanced‐stage cHL patients aged 60 years or more (ClinicalTRials.Gov identifier 02467946).

## PATIENTS AND METHODS

### Study description

Advanced‐stage (IIB‐IVB) patients aged 60–80 years, diagnosed with cHL according to the World Health Organization (WHO), were recruited across five Italian and five French participating centres. The enrolled patients had to fulfil all the following requirements: no previous treatment, good performance status (PS: Eastern Cooperative Oncology Group [ECOG] ≤2), adequate hemometry and organ function as follows: (1) absolute neutrophil count (ANC) ≥1.5 × 10^9^/L; (2) haemoglobin ≥9 g/dL; (3) platelets (PTL) ≥100 × 10^9^/L; (4) aspartate aminotransferase–alanine aminotransferase ≤2.5 × upper limit of normal (ULN); (5) bilirubin ≤1.5 × ULN; (6) creatinine <150 μmol/L (or 1.7 mg/dL), a good cardiac performance, documented by left ventricular ejection fraction (LVEF) >50%. After a complete baseline staging according to Lugano 2014 recommendations,^16^ patients underwent geriatric assessment with activity of daily living (ADL), instrumental activities of daily living (IADL) and Cumulative Illness Rating Scale (CIRS‐G).^17^ Treatment consisted of six cycles of the Be‐BV combination with the following schedule: BV was administered at the dose of 1.2 mg/kg, as an intravenous infusion (i.v.) over 30 min. One hour after BV infusion, Be was administered at the dose of 90 mg/m^2^/day, for 2 days by i.v. injection over 30–60 min. The Be‐BV combination was administered every 3 weeks for a total of six courses, and an interim positron emission tomography (PET) (PET‐2) was performed after two Be‐BV cycles. Patients with a positive PET‐2 continued with two more Be‐BV cycles and a second interim PET (PET‐4) was performed. If the latter was still positive, patients were withdrawn from the study and underwent a rescue treatment. Patients with a negative PET‐2 continued the planned treatment. Patients were followed for up to 6 years to assess long‐term outcomes.

### Study design

The primary study end‐point during phase 1 was safety, and during phase 2, efficacy. Upon inclusion of 60 patients, both parameters had to be assessed in a two‐phase study, according to Simon^18^:

Sample size calculation was performed in sample size table for clinical studies software version 1.0 (D. Machin) (Figure S1).

To confirm the results of local evaluations, a blinded independent central review (BICR) for PET‐2 scans was implemented. PET images were uploaded on the study website and subsequently transferred via the WEB‐based Imaging Diagnosis by an Expert Network platform® to two nuclear imaging experts, as previously described, for BICR.^19^, ^20^ The interpretation key adopted was a visual assessment, according to the 5‐point Deauville scale (5P‐S),^21^ and the interim response assessment was scored according to Lugano 2014 criteria.^22^

### Statistical considerations

All the analyses were done according to the intention‐to‐treat principle (ITT) and in a per‐protocol (PP) analysis. Baseline continuous characteristics were presented as a median value with range, and compared using the Mann–Whitney *U* test. Categorical variables were reported with absolute and percentage frequencies and compared with chi‐squared test or Fisher's exact test. Survival outcomes were estimated using the Kaplan–Meier method, and the log‐rank test was applied to test differences between groups. All the patients were included in an ITT and PP in all survival analysis. The PP population was defined as a patient cohort treated with all the six cycles of treatment, without major deviation. The treatment outcome measures were defined according to the revised response criteria for malignant lymphoma.^22^ PFS was measured from the date of trial registration to the date of first evidence of disease progression, relapse or death from any cause. OS was measured from the date of registration to the date of death from any cause. Univariable and multivariable Cox proportional sub‐distribution hazard regression models were also fit to check for variables affecting PFS. For multivariable analysis factors with *p* ≤ 0.05 on univariable analysis were entered.

Detailed severe adverse effects (SAEs) were recorded with National Cancer Institute Common Terminology Criteria (NCI‐CTC) for adverse events, version 4.0. Analyses were conducted with R, version 4.3.1.

### Sample size calculation

The Optimum Simon two‐stage design^18^ was applied for sample size calculation. The following hypotheses were made for complete response (CR) rate:

- P0 has been taken as 0.60 (uninteresting level)
- P1 has been taken as 0.80 (desirable target level)
- Alpha = 0.05
- Power = 90%

Under these hypotheses, 53 patients had to be included and treated with the Be‐BV combination. A two‐stage Simon design was utilized to conduct the study as follows: in the first stage an evaluation was performed after inclusion of 19 patients with the following stopping rules: (1) If <13/19 evaluable patients were in CR, the trial had to be stopped with the conclusion that the therapeutic activity of the combination is too low; (2) If ≥13/19 evaluable patients were in CR, the trial had to be continued with inclusion of 34 more patients; (3) If more than 6/19 patients experienced toxicity events, as defined in the stopping rules, Independent Data Monitoring Committee had to validate it and the study had to be stopped. In the second stage after inclusion of 53 patients the trial could be stopped if: (1) <38/53 evaluable patients were in CR, (the therapeutic activity Be‐BV is too low); (2) If ≥38/53 evaluable patients were in CR, (the therapeutic activity of Be‐BV is adequate).

Considering 10% of patients falsely included, not assessable or showing a major protocol deviation, a total of 60 patients had to be included in the study.

## RESULTS

Since July 2015 until February 2019, a total of 63 patients were screened. Of these, 60 were eligible and enrolled across five centres in France and five in Italy. In 59 out of 60 cases the diagnostic biopsy blocs were available for review; one bloc was missing, but the patient was retained in the study. The central review confirmed the diagnosis of cHL according to WHO in 58 out of 59, with the following histotype breakdown: nodular sclerosis 32, mixed‐cellularity 9, lymphocyte‐rich 5, not otherwise specified (NOS) 12. One patient whose histological review proved consistent with angioimmunoblastic T‐cell lymphoma was excluded from the analysis. One patient never initiated therapy, and another received only one treatment cycle. As a result, 57 patients were evaluable for the primary end‐point (Figure 1).

**FIGURE 1:**
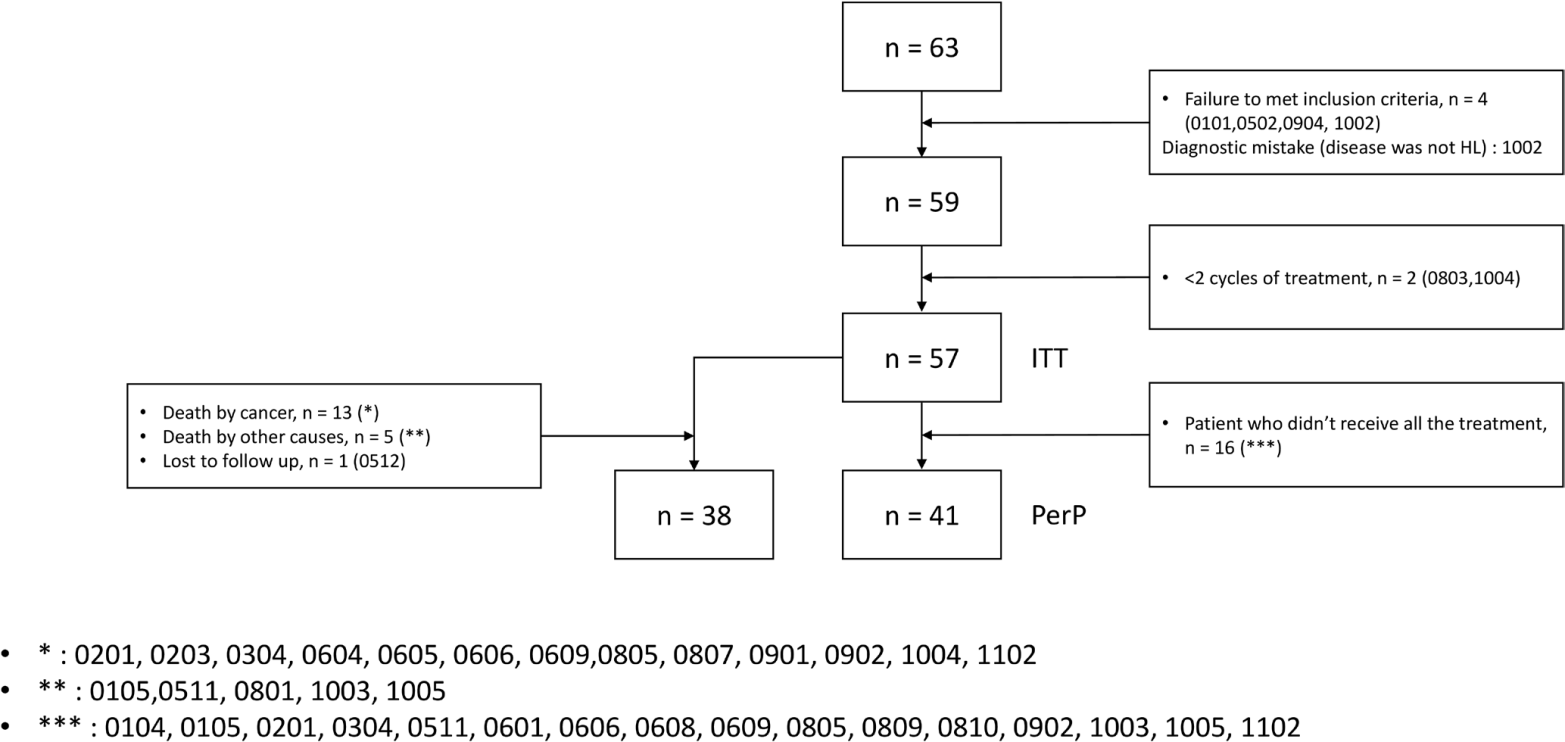
Patient flowchart.

### Demographics

Demographics and clinical characteristics of the evaluable patients is depicted in Table 1. The mean age was 71 (62–79), and M/F ratio 40/17. The Ann‐Arbor stage was IIB in 12, III in 14 and IV in 31 and B‐symptoms were recorded in 39. IPS was 0–1 in 2, 2–3 in 26 and >3 in 29; PS was 0–1 in 51 and 2 in 6, while 68% of patients had ≥5 comorbidities. Despite an ADL score ≥6 in 85% and a score IADL of 8 in 77% of them, showing a good functional ability in the daily living activity, the CIRS‐G questionnaire for co‐morbidity,^17^, ^23^ performed in 52/57 (91%) and 39/52 (75%) showed a score >3 (organ impairment requiring immediate treatment) with a median score of 11.5 (0–22). Among the 57 patients included in the analysis, a total of 147 comorbidities were recorded, consisting mostly of cardiovascular disease (45/147), metabolism disorders (35/147), gastrointestinal disorders (12/147) and reproductive system and breast disorders (12/147).

**TABLE 1:** Demographics and clinical characteristics of the enrolled patients.

Modality	Missing		
Age			
Mean (range)	0	70.75 (62–79)	
Gender			
M/F	0	40/17	
Stage			
IIB	0	12 (21%)	
III		14 (24%)	
IV		31 (55%)	
Performance status (ECOG)			
0	0	26 (46%)	
1		25 (44%)	
2		6 (10%)	
B‐symptoms			
No	0	18 (32%)	
Yes		39 (68%)	
IPS (Hasenclever)			
0–1	0	2 (3.5%)	
2–3		26 (45.6%)	
>3		29 (50.9%)	
Nodal mass >10 cm.			
No	0	54 (95%)	
Yes		3 (5%)	
Haemoglobin			
Mean (gr/dL)	0	12.07	
min‐1stq‐med‐3rdq‐max		7.6‐10.6‐12.1‐13.6‐16.1	
White blood cells			
Mean (n/μL)	3	9.76	
min‐1stq‐med‐3rdq‐max		3.88‐6.26‐8.25‐13.53‐19.72	
Lymphocytes			
Mean (n/μL)	3	1.34	
min‐1stq‐med‐3rdq‐max		0.2‐0.92‐1.32‐1.8‐2.9	
Neutrophils			
Mean (n/μL)	3	7.29	
min‐1stq‐med‐3rdq‐max		2.12‐4.08‐5.9‐10.74‐17.85	
Albumin			
Mean (gr/dL)	40	4.86	
min‐1stq‐med‐3rdq‐max		3.1‐3.6‐4.‐4.43‐19.7	
LDH (NV)			
<UNV	0	43	
>UNV		14	
Co‐morbidity			
0		4	
1		13	
2		14	
3		10	
4		8	
5		6	
>5		2	
CIRS			
Mean	5	10.63	
Quartiles		0‐3.75‐11.50‐17‐22	
ADL			
Modality/N° (Frequency)	4	3	1 (1.9%)
		4	1 (1.9%)
		4.5	1 (1.9%)
		5	3 (5.7%)
		5.5	2 (3.8)
		6	45 (84.9%)
IADL			
Modality/N° (Frequency)	5	2	1 (1.9%)
		4	2 (3.8%)
		5	2 (3.8%)
		6	1 (1.9%)
		7	6 (11.5%)
		8	40 (76.9%)

Abbreviations: LDH: Lactate dehydrogenase; NV: Normal Value; UNV: Upper Normal Value.

### Toxicity

Of 57 evaluable patients, 41 (72%) completed the treatment according to the pre‐planned therapeutic schedule. Sixteen did not complete the treatment for the following reasons (Table 2): treatment failure (6), acute toxicity or treatment delay >2 weeks because of persisting cytopenia (10).

**TABLE 2:** Treatment outcome.

Variable	Total	NA
Total	16	
Reason to stop treatment		0 (0%)
Failure	6 (37.5)	
Toxicity	10 (62.5)	
Reason for treatment discontinuation		0 (0%)
Less than PR	2 (12.5)	
Progression during treatment (2/4 CMV positive)	4 (25)	
Repeated toxicity (CMV positive)	1 (6.2)	
Death related to HL	1 (6.2)	
Treatment delay >15 days (4/7 CMV positive)	7 (43.8)	
Undefined	1 (6.2)	
CMV prevalence		0 (0%)
No	9 (56.2)	
Yes	7 (43.8)	
Final outcome		0 (0%)
ALIVE at 75 months of FU	34 (60%)	
Death related to Hodgkin lymphoma	8 (14%)	
Death other cause	15 (26%)	

Abbreviations: ALIVE, FU: Patient alive at the end of treatment addressed to clinical follow‐up.

During treatment, non‐infectious toxicity, in terms of WHO grade 3 or more severe adverse effect (SAE), consisted in lymphopenia (29.9%), neutropenia (19.36%), leucopenia (2.8%), thrombocytopenia (1.9%), infusion‐related reactions (1.9%) and cutaneous disorders (4.7%) (Table S1). Overall, the infectious toxicity consisted in a clinically documented cytomegalovirus (CMV) infection (5.6%), while febrile neutropenia was recorded in three chemotherapy cycles (2.8%). Interestingly, CMV reactivation was observed in 18 (32%) patients (Table 3): 13 were treated with valganciclovir and 5 did not receive any treatment. Among these 18 patients, 3 died with CMV viraemia. Importantly, the higher prevalence of CMV reactivation in the Italian cohort was likely due to a difference in screening practices across both countries: CMV detection (by polymerase chain reaction (PCR) or viral load measurement) was not mandatory at baseline for study inclusion. However, in most Italian centres, CMV testing was performed systematically during follow‐up, which may have led to a higher number of identified reactivations compared to centres where testing was performed only on clinical suspicion. Twenty‐five patients out of 57 (44%) have died, the causes of death being recurrent cHL (8), sepsis (1), unknown toxicity (1), secondary malignancy (3), respiratory failure (1) and unknown (11). Among them, 16 were in disease progression or relapse and 8 developed a second cancer (Figure 2).

**TABLE 3:** Cytomegalovirus (CMV) reactivation description.

SUBJID	Concurrent (during treatment period) or after treatment	Onset grade of the adverse event	Action taken with treatment	Ongoing at death		Treatment	Indication
0511	Concurrent (After C2)	3	No modification	No	Treated	CYMEVAN	CMV
0609	Concurrent (After C5)	2	No modification	No	Untreated	ACICLOVIR	Prophylaxis
0701	Concurrent (After C6)	1	No modification	No	Treated	VAGANCICLOVIR	CMV
0702	Concurrent (After C5)	2	No modification	No	Treated	VAGANCICLOVIR	CMV
0703	Concurrent (After C4)	2	Course delayed	No	Treated	VAGANCICLOVIR	CMV
0801	Concurrent (After C5)		Course delayed	No	Treated	VAGANCICLOVIR	
0802	Concurrent (After C2)	2	Course delayed	No	Treated	VAGANCICLOVIR	CMV
0804	Concurrent (After C1)	3	No modification	No	Treated	VAGANCICLOVIR	CMV
0805	Concurrent (After C3)	2	Permanent discontinuation	YES	Treated	VAGANCICLOVIR	CMV
0807	Concurrent (After C4)	2	Course delayed	No	Treated	VAGANCICLOVIR	CMV
0808	Concurrent (After C3)	2	No modification	No	Treated	VAGANCICLOVIR	CMV
0809	Concurrent (After C4)	2	No modification	No	Untreated	ACICLOVIR	Prophylaxis
0810	Concurrent (After C3)	3	Permanent discontinuation	No	Treated	VALGANCICLOVIR FOSCARET GANCICLOVIR	CMV
0902	Concurrent (After C2)	2	Course delayed	No	Treated	VAGANCICLOVIR	VALCYTE
1003	Concurrent (After C4)	2	Permanent discontinuation	NO	Untreated	ACICLOVIR	Prophylaxis
1004	After (After C1)	2	NA (discontinuation for disease progression)	YES	Untreated	ACICLOVIR	Prophylaxis
1005	Concurrent (After C2)	1	Course delayed	YES	Treated	VAGANCICLOVIR	CMV
1006	Concurrent (After C2)	1	Course delayed	NO	Treated	VAGANCICLOVIR	CMV

**FIGURE 2:**
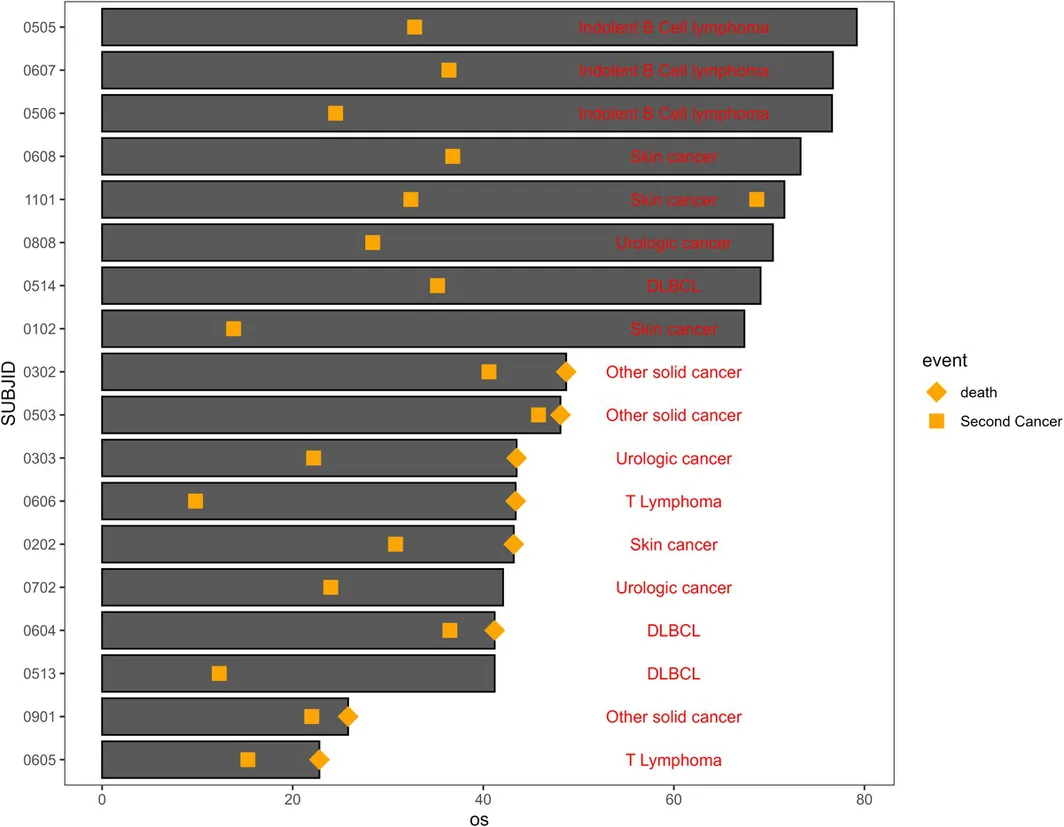
Secondary cancer and overall survival.

The safety end‐point of the study was met as <3 and <4 patients experienced toxicities during phase 1 and phase 2 respectively.

### Efficacy

All 57 patients but 3 underwent an interim treatment evaluation with PET after two Be‐BV courses (PET‐2); the reasons for omitting PET‐2 were: investigator decision (1), disease progression (1) and unknown reason (1).

In an intention‐to‐treat (ITT) analysis, 45 out of 57 evaluable patients (79%) achieved a complete metabolic response (CMR) in End‐of‐Treatment (EOT)‐PET. The end‐point of effectiveness, although not strictly defined in terms of CMR rate, was met. On the other hand, in a per‐protocol (PP) analysis, 35 out of 40 patients (87.5%) who completed the treatment with six Be‐BV cycles achieved a CMR, four showed disease relapse, one patient had a response loss. In PET‐2, CMR was recorded in 45 out of 54 patients (83.3%) in the ITT and in 34 out of 41 patients (82.9%) in the PP analysis. Among patients who did not achieve a CR in PET‐2, 9 out of 18 (50.0%) in the ITT and 8 out of 12 (66.7%) in PP analysis achieved a CMR by cycle 4 (Figure S4 and Table S3). The end‐point of effectiveness was met as most patients (79%) achieved a CMR at the end of treatment.

For the five patients with progressive disease, the median time to progression from diagnosis was 3.4 (0.8–4.7) months. For the 17 relapsing patients the median time for relapse from diagnosis was 11.5 (3.1–28.9) months.

The 6‐y OS and PFS in ITT analysis were 56% (95% confidence intertvals [CI] 44–72) and 36% (95% CI 25–52) (Figures S2 and S3). The 6‐y OS and PFS in PP analysis were 67% (95% CI 53–84) and 45% (95% CI 32–65) respectively (Figures 3 and 4). Briefly, 35 patients had a PFS event: 13 progressions (10 deaths), 13 relapses (6 deaths) and 9 deaths unrelated to cHL.

**FIGURE 3:**
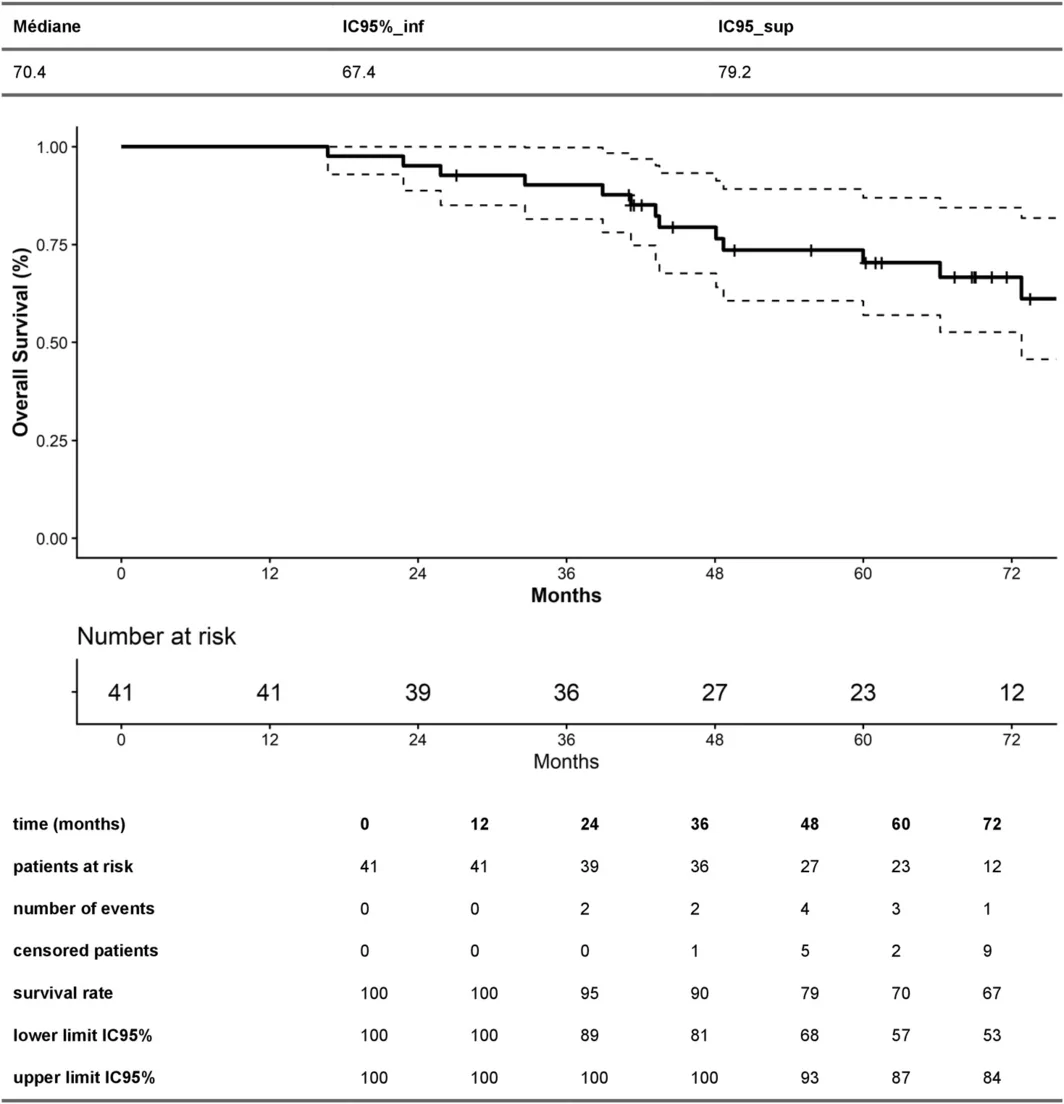
Overall survival per‐protocol analysis.

**FIGURE 4:**
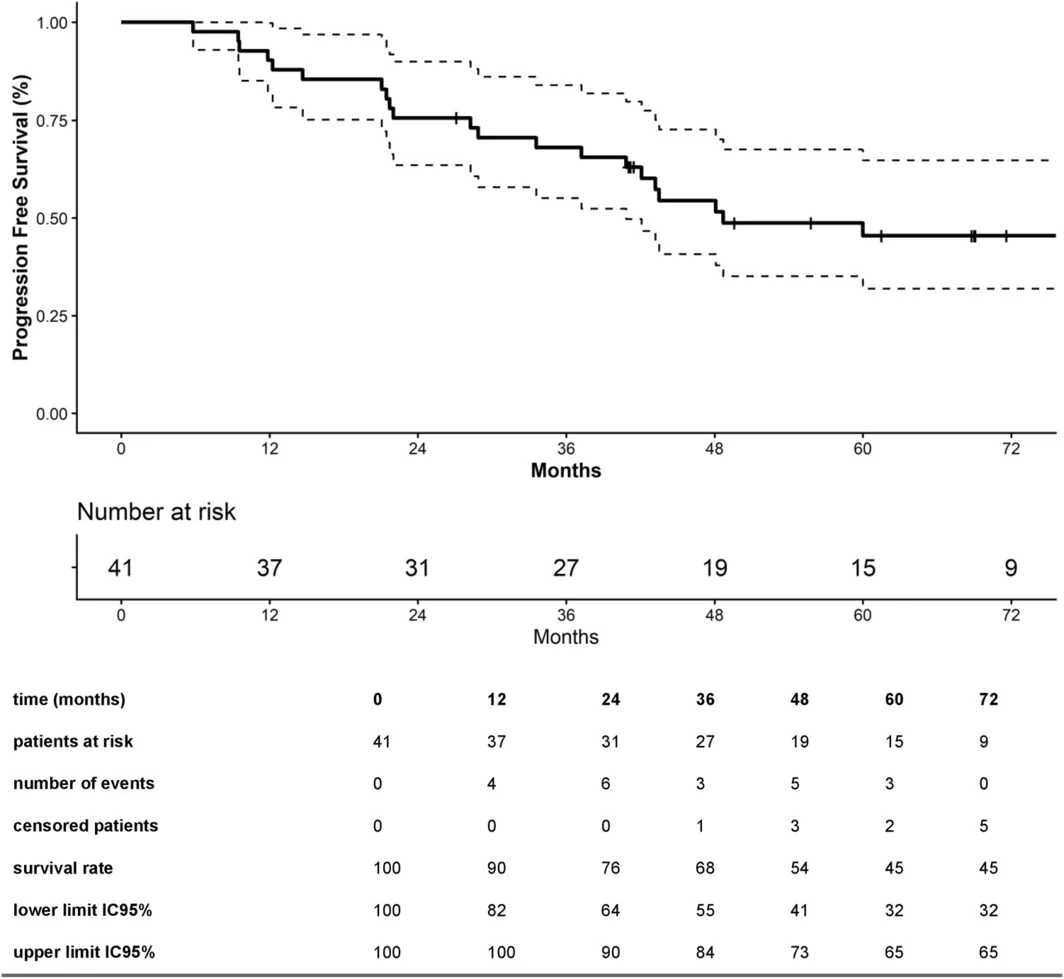
Progression‐free survival per‐protocol analysis.

## DISCUSSION

The treatment of cHL in the elderly still represents an unmet clinical need. The open‐label, phase II, multicentre HALO trial confirmed the effectiveness of Be‐BV combination in first‐line treatment of Hodgkin lymphoma (HL) in elderly unfit patients with unfavourable prognostic factors. Most patients included in the study presented with advanced‐stage (III and IV in 79%), a high IPS score (>3 in 51%) and a high co‐morbidity rate, with a median of 10.63 concurrent pathological conditions. Although an anthracycline‐free treatment proved less efficacious compared to the doxorubicin‐based regimens for the treatment of cHL in the elderly,^2^, ^12^, ^16^, ^24^ we decided to explore safety and effectiveness of the frontline use of Be‐BV combination in this high‐risk patient subset. This regimen proved able to induce disease remission in up to 73% of the relapsed/refractory cHL patients.^13^ Despite a good PS and a high ADL and IADL test recorded in up to 90% of the patients, the CIRS‐G score, an independent predictor of a poor survival in elderly cancer patients,^23^, ^25^, ^26^ showed a consistent organ impairment in 66% of the enrolled patients. In summary, the following adverse predictors of treatment outcome recorded in HALO trial were: (1) an anthracycline‐free regimen; and (2) a CIRS‐G score for organ impairment indicating a relevant co‐morbidity in 70% of the patients, requiring an immediate treatment in 27%. Both tests would have classified those patients unfit and frail according to the classical definition of Tucci et al.^25^

The high CR rate in PP (35/41) of 85% observed in the present study confirms the optimal immediate disease control achieved by BV monotherapy in untreated^14^ and relapsed^27^ elderly cHL patients. After a prolonged median follow‐up of 69.1 (67.4–77.2) months, the PFS rate was 45% (95% CI 32–65) vs. 36% (95% CI 25–52) in PP and ITT analysis, respectively, with all the PFS events recorded after the end of treatment (21 events in PP vs. 35 events in ITT). This difference of treatment outcome was mainly due to a reduction in the dose intensity of Be‐BV treatment in 16 patients. In summary, the reason for treatment discontinuation was treatment failure in 6/57 (10.5%) and 10/57 (17.5%) for toxicity including CMV reactivation with/out persisting cytopenia and fever (Table S2).

CMV reactivation is a rather common complication in patients affected by lymphoproliferative disorders treated with Be and showing a long‐lasting post‐chemotherapy lymphocytopenia.^28^ Picardi et al. reported the effectiveness of a rescue treatment (combination of BV 1.8 mg/Kg. in day 1 followed by high‐dose Be, 120 mg/m^2^ on days 2 and 3) to bridge relapsed/refractory HL patients after a median of 3 chemotherapy cycles to autologous haematopoietic stem cell transplantation^29^. Twenty‐patients were studied, 10 (50%) developed persistent leucopenia and 5 (25%) developed CMV reactivation with viraemia (median CMV‐Deoxy‐riboNucleic Acid (DNA) 1810 IU/mL), with a prompt cytopenia restoration upon valganciclovir treatment. Therefore, CMV‐DNA monitoring should be considered mandatory during the Be‐BV regimen.

The present study seems to confirm the efficacy of Be‐BV combined treatment in the PP cohort of patients having completed the full pre‐planned 6 cycles of Be‐BV, with a 2‐Y PFS remaining at 76% (95% CI 64–90). Taking into account that the study HALO adopted an anthracycline‐free regimen with a treatment duration shorter than 5 months (126 days), those results compare favourably with that reported in the sequential association of BV and AVD anthracycline‐containing regimen proposed by Evens et al.,^14^ with a duration of treatment spanning twice compared to HALO trial (252 days). On the other hand, the 6‐Y PFS of 45% obtained in HALO (95% CI 32–65) was definitely lower compared to 66% (95% CI 54–76) reported in the ECHELON‐1 trial.^14^ However, the remarkable difference of demographic and prognostic factors between ECHELON‐1 and HALO makes a comparison between these two studies unfeasible. Moreover, it is worth noting that HALO trial specifically targeted a less fit population than that of the ECHELON‐1 trial. Consequently, the dose of BV was intentionally adapted from 1.8 mg/kg to 1.2 mg/kg, to account for this frailty and to optimize the safety profile. In fact, the ECHELON‐1 trial enrolled a young and fit population, with only 57 (14% of the total enrolled population) aged >60 years, and in this age subset the prevalence of grade ≥3 AEs was recorded in 50 patients (88%), higher than that reported in younger patients. In this fraction of patients, the 5‐years PFS was 67.1% and there was not a significant superiority compared then that reported with the ABVD (61.6%).^14^ In conclusion, the results of the HALO trial, while stressing the pitfalls of proposing a ‘standard approach’ for the treatment of elderly unfit patients, for whom a Be‐BV could be a reasonable alternative, confirm that dose intensity and doxorubicin‐based regiments remain an essential goal in treating cHL in the elderly.

## AUTHOR CONTRIBUTIONS

**Tanguy Pace‐Loscos:** Formal analysis; visualization; writing – review and editing. **Simonetta Viviani:** Investigation; writing – review and editing. **Caterina Patti:** Investigation. **Renaud Schiappa:** Software; data curation. **Alessandro Rambaldi:** Investigation. **Anna Borra:** Investigation. **Pierre Feugier:** Investigation. **Cécile Borel:** Investigation. **Anna Guidetti:** Investigation. **Fontanet Bijou:** Investigation. **Colin Debaigt:** Project administration; writing – review and editing. **Olivier Humbert:** Investigation. **Jean‐Marc Schiano De Colella:** Investigation; writing – original draft. **Andrea Gallamini:** Conceptualization; investigation; funding acquisition; writing – original draft; writing – review and editing. **Stephane Chauvie:** Software; data curation. **Davide Rapezzi:** Investigation.

## FUNDING INFORMATION

This investigator‐sponsored study was supported by Millenium Pharmaceuticals, which provided financial funding and the study drug brentuximab vedotin at no cost. While the funder was provided with the opportunity to review the manuscript for medical accuracy and to ensure the protection of proprietary information prior to submission, all the editorial decisions and the final content remained entirely under the control of the authors. The funder had no role in the study design, data collection or data analysis.

## CONFLICT OF INTEREST STATEMENT

Andrea Gallamini reports receiving research grants and study drug from Millenium/TAKEDA for the submitted work and personal fees for consultancy and speaker bureau from TAKEDA outside the submitted work. Simonetta Viviani reports receiving funds for attending meetings from TAKEDA. Bijou Fontanet reports receiving funds for attending meetings from TAKEDA. All other authors have no relevant financial or non‐financial interests to disclose.

## ETHICS STATEMENT

The study was approved by the national ethic committee in France (Sud Mediterranean V, 9 June 2015, reference 15.041) and in Italy (Istituto Superiore di Sanità, 16 January 2017, reference ISS N°: 08231(15)CMS.21‐1344).

## PATIENT CONSENT STATEMENT

All patients enrolled in the study signed an informed consent. Original documents will be available on request.

## CLINICAL TRIAL REGISTRATION

ClinicalTRials.Gov identifier: 02467946, Eudract number: 2014‐003320‐51.

## Supporting information


**Figure S1.** Phase I/II study design.
**Figure S2.** Overall survival intention‐to‐treat analysis.
**Figure S3.** Progression‐free survival intention‐to‐treat analysis.
**Figure S4.** Patient flow according to PET assessments.
**Table S1.** Grade 3 or higher toxicities related to BV‐Be treatment.
**Table S2.** Reasons for treatment discontinuation and subsequent outcomes.
**Table S3.** Complete response rates by cycle and population.

## Data Availability

The data that support the findings of this study are available from the corresponding author upon reasonable request.
