# Supplementary material for: Long‐term results of brentuximab vedotin and bendamustine for first‐line treatment of Hodgkin lymphoma in the elderly (HALO trial)
Source: Br J Haematol. 2026 Aug 26;209(4):1619–28. doi: 10.1111/bjh.70763 (PMC13674205; doi:10.1111/bjh.70763)
Supplement: Supplementary file 1 [file BJH-209-1619-s001.docx]

Brentuximab-Vedotin and Bendamustine is an active drug combination for first-line treatment of Hodgkin lymphoma in the elderly patient with co-morbidity (HALO trial): long-term results.

**Short Title**

Brentuximab Vedotin & Bendamustine in elderly HL

Supplemental figures and tables :

Supplemental Figure S1 : Phase I/II Study design

Supplemental Figure S2 : Overall Survival Intention To Treat analysis

Supplemental Figure S3 : Progression Free Survival Intention To Treat analysis

Supplemental Figure S4 : Patient flow according to PET assessments

Supplemental Table S1: Grade 3 or Higher Toxicities Related to BV-Be Treatment

Supplemental Table S2: Reasons for Treatment Discontinuation and Subsequent Outcomes

Supplemental Table S3: Complete Response Rates by Cycle and Population

Supplemental Figure S1 : Phase I/II Study design

Supplemental Figure S2 : OS ITT

| **Médiane** | **IC95%_inf** | **IC95_sup** |
| --- | --- | --- |
| 69.1 | 67.4 | 77.2 |

| **time (months)** | **0** | **12** | **24** | **36** | **48** | **60** | **72** |
| --- | --- | --- | --- | --- | --- | --- | --- |
| **patients at risk** | 57 | 55 | 47 | 43 | 31 | 27 | 14 |
| **number of events** | 0 | 2 | 8 | 3 | 6 | 3 | 1 |
| **censored patients** | 0 | 0 | 0 | 1 | 6 | 2 | 11 |
| **survival rate** | 100 | 96 | 82 | 77 | 65 | 59 | 56 |
| **lower limit IC95%** | 100 | 92 | 73 | 67 | 54 | 47 | 44 |
| **upper limit IC95%** | 100 | 100 | 93 | 89 | 79 | 74 | 72 |

Supplemental Figure S3 : PFS ITT

| **n.max** | **events** | **median** | **0.95LCL** | **0.95UCL** |
| --- | --- | --- | --- | --- |
| 57 | 35 | 40.83 | 22.01 |  |

| **time (months)** | **0** | **12** | **24** | **36** | **48** | **60** | **72** |
| --- | --- | --- | --- | --- | --- | --- | --- |
| **patients at risk** | 57 | 47 | 35 | 30 | 21 | 17 | 10 |
| **number of events** | 0 | 10 | 12 | 4 | 6 | 3 | 0 |
| **censored patients** | 0 | 0 | 0 | 1 | 3 | 2 | 6 |
| **survival rate** | 100 | 82 | 61 | 54 | 43 | 36 | 36 |
| **lower limit IC95%** | 100 | 73 | 50 | 43 | 31 | 25 | 25 |
| **upper limit IC95%** | 100 | 93 | 75 | 69 | 58 | 52 | 52 |

Supplemental Figure S4: Patient flow according to PET assessments

Supplemental Table S1: Grade 3 or Higher Toxicities Related to BV-Be Treatment

| **System Organ Class** | **Term** | **Grade** | **Total** |
| --- | --- | --- | --- |
| BLOOD AND LYMPHATIC SYSTEM DISORDERS | ANAEMIA | 3 | 2 (1.87 %) |
|  | FEBRILE NEUTROPENIA | 3 | 3 (2.8 %) |
|  | LEUKOPENIA | 3 | 2 (1.87 %) |
|  |  | 4 | 1 (0.93 %) |
|  | LYMPHOPENIA | 4 | 19 (17.76 %) |
|  |  | 3 | 13 (12.15 %) |
|  | NEUTROPENIA | 3 | 17 (15.89 %) |
|  |  | 4 | 4 (3.74 %) |
|  | THROMBOCYTOPENIA | 3 | 2 (1.87 %) |
| CARDIAC DISORDERS | BRADYCARDIA | 3 | 1 (0.93 %) |
| GASTROINTESTINAL DISORDERS | STOMATITIS | 3 | 2 (1.87 %) |
| GENERAL DISORDERS AND ADMINISTRATION SITE CONDITIONS | DEATH | 5 | 1 (0.93 %) |
|  | FATIGUE | 3 | 1 (0.93 %) |
|  | GENERAL PHYSICAL HEALTH DETERIORATION | 3 | 1 (0.93 %) |
|  | PYREXIA | 3 | 1 (0.93 %) |
| IMMUNE SYSTEM DISORDERS | HYPERSENSITIVITY | 3 | 1 (0.93 %) |
| INFECTIONS AND INFESTATIONS | BREAST CELLULITIS | 3 | 1 (0.93 %) |
|  | CYTOMEGALOVIRUS INFECTION | 3 | 4 (3.74 %) |
|  | CYTOMEGALOVIRUS INFECTION REACTIVATION | 3 | 2 (1.87 %) |
|  | DEVICE RELATED INFECTION | 3 | 1 (0.93 %) |
|  | ESCHERICHIA URINARY TRACT INFECTION | 3 | 1 (0.93 %) |
|  | OTITIS EXTERNA BACTERIAL | 3 | 1 (0.93 %) |
|  | PROSTATE INFECTION | 3 | 1 (0.93 %) |
| INJURY, POISONING AND PROCEDURAL COMPLICATIONS | INFUSION RELATED REACTION | 3 | 2 (1.87 %) |
| INVESTIGATIONS | ALANINE AMINOTRANSFERASE INCREASED | 3 | 1 (0.93 %) |
|  | GAMMA-GLUTAMYLTRANSFERASE INCREASED | 3 | 2 (1.87 %) |
|  | INTERNATIONAL NORMALISED RATIO INCREASED | 3 | 1 (0.93 %) |
| METABOLISM AND NUTRITION DISORDERS | CELL DEATH | 3 | 1 (0.93 %) |
|  | DECREASED APPETITE | 3 | 1 (0.93 %) |
| NEOPLASMS BENIGN, MALIGNANT AND UNSPECIFIED (INCL CYSTS AND POLYPS) | ACUTE MYELOID LEUKAEMIA | 5 | 1 (0.93 %) |
|  | DIFFUSE LARGE B-CELL LYMPHOMA | 3 | 1 (0.93 %) |
|  | NODAL MARGINAL ZONE B-CELL LYMPHOMA | 4 | 1 (0.93 %) |
|  | NON-HODGKIN'S LYMPHOMA | 5 | 1 (0.93 %) |
| NERVOUS SYSTEM DISORDERS | DYSGEUSIA | 3 | 1 (0.93 %) |
|  | NEUROPATHY PERIPHERAL | 3 | 1 (0.93 %) |
|  | PRESYNCOPE | 3 | 1 (0.93 %) |
| RESPIRATORY, THORACIC AND MEDIASTINAL DISORDERS | LUNG DISORDER | 3 | 1 (0.93 %) |
|  | PNEUMONITIS | 4 | 1 (0.93 %) |
|  | PULMONARY EMBOLISM | 3 | 1 (0.93 %) |
| SKIN AND SUBCUTANEOUS TISSUE DISORDERS | ERYTHEMA | 3 | 1 (0.93 %) |
|  | PRURITUS | 3 | 1 (0.93 %) |
|  | RASH | 3 | 3 (2.8 %) |
|  | RASH MACULO-PAPULAR | 3 | 1 (0.93 %) |
| VASCULAR DISORDERS | HYPOTENSION | 3 | 1 (0.93 %) |

Supplemental Table S2: Reasons for Treatment Discontinuation and Subsequent Outcomes

| **SUBJID** | **Reason for treatment discontinuation** | **Toxicity (grade)** | **BE dose reduction** | **BV dose reduction** | **concomitant treatment** | **Final outcomes** | **os** |
| --- | --- | --- | --- | --- | --- | --- | --- |
| 0104 | TREATMENT FAILURE |  | NO | NO |  | Alive | 40.40 |
| 0105 | TREATMENT DELAYED MORE THAN TWO WEEKS FOLLOWING TOXICITY | SEPSIS (3) | NO | NO | TAZOCILLINE + AMIKACINE + VANCOMYCINE + CIFLOX | Death unknown cause | 17.38 |
| 0201 | UNACCEPTABLE TOXICITY | ALLERGIC REACTION (3) | YES | NO | POLARAMINE + SOLUMEDROL + PERFALGAN + BROMAZEPAM | Death related to Hodgkin lymphoma | 14.36 |
| 0304 | DISEASE PROGRESSION |  | YES | YES |  | Death related to Hodgkin lymphoma | 22.21 |
| 0511 | UNACCEPTABLE TOXICITY | HEPATIC CYTOLISIS (4) + CHOLESTASIS HEPATITIS (4) | NO | NO |  | Death unknown cause | 15.01 |
| 0601 | DISEASE PROGRESSION |  | NO | NO |  | Death | 75.20 |
| 0606 | TREATMENT FAILURE |  | YES | YES |  | Death related to treatment toxicity | 43.40 |
| 0608 | TREATMENT DELAYED MORE THAN TWO WEEKS FOLLOWING TOXICITY | THROMBOCYTOPENIA (2) | YES | YES |  | Alive | 73.30 |
| 0609 | TREATMENT DELAYED MORE THAN TWO WEEKS FOLLOWING TOXICITY | THROMBOCYTOPENIA (2) | YES | NO |  | Death related to Hodgkin lymphoma | 13.96 |
| 0805 | DISEASE PROGRESSION |  | NO | YES |  | Death related to Hodgkin lymphoma | 4.01 |
| 0809 | DISEASE PROGRESSION |  | YES | YES |  | Alive | 69.10 |
| 0810 | TREATMENT DELAYED MORE THAN TWO WEEKS FOR ANY EVENT | LYMPHOCYTOPENIA (4) | NO | NO |  | Alive | 64.10 |
| 0902 | RECURRENCE OF TOXICITY OCCURRENCE AFTER DOSE REDUCTION OF BE OR BV | CMV INFECTION (2) + LYMPHOCYTE COUNT DECREASE (3) + NEUTROPENIA (2) + THROMBOCYTOPENIA (2) | YES | NO | VALCYTE + VALGANCICLOVIR (CMV INFECTION) | Death related to Hodgkin lymphoma | 13.53 |
| 1003 | TREATMENT DELAYED MORE THAN TWO WEEKS FOR ANY EVENT | CMV REACTIVATION (2) + PNEUMONITIS (4) | YES | YES | ANTIBIOTIC THERAPY + STEROID THERAPY (PNEUMONITIS) | Death unknown cause | 40.54 |
| 1005 | TREATMENT DELAYED MORE THAN TWO WEEKS FOR ANY EVENT | CMV INFECTION (2) + EBV INFECTION (2) | NO | NO | VALGANCICLOVIR (CMV INFECTION) | Death unknown cause | 2.30 |
| 1102 | UNACCEPTABLE TOXICITY | PARESTHESIA (2) | YES | NO | BACTRIM | Death related to Hodgkin lymphoma | 28.19 |

Supplemental Table S3: Complete Response Rates by Cycle and Population

| Deauville Scoring by Centralized Review | **Response** | **Number** | **Frequence** | **NA** |
| --- | --- | --- | --- | --- |
| End of treatment ITT |  |  |  | 1 (1.75%) |
|  | COMPLETE RESPONSE | 45 | 80.36% |  |
|  | FAILURE | 11 | 19.64% |  |
| End of treatment PP |  |  |  | 1 (2.44%) |
|  | COMPLETE RESPONSE | 35 | 87.5% |  |
|  | PARTIAL RESPONSE | 1 | 2.5% |  |
|  | PROGRESSIVE DISEASE | 4 | 10% |  |
| End of cycle 2 itt |  |  |  | 0 (0%) |
|  | COMPLETE RESPONSE | 45 | 78.95% |  |
|  | FAILURE | 12 | 21.05% |  |
| End of cycle 2 PP |  |  |  | 0 (0%) |
|  | COMPLETE RESPONSE | 34 | 82.93% |  |
|  | FAILURE | 7 | 17.07% |  |
| End of cycle 4 itt |  |  |  | 0 (0%) |
|  | COMPLETE RESPONSE | 9 | 50% |  |
|  | FAILURE | 9 | 50% |  |
| End of cycle 4 PP |  |  |  | 0 (0%) |
|  | COMPLETE RESPONSE | 8 | 66.67% |  |
|  | FAILURE | 4 | 33.33% |  |
